# COVID-19 information dissemination in Uganda: Perspectives from sub-national health workers

**DOI:** 10.1186/s12913-021-07068-x

**Published:** 2021-10-07

**Authors:** Cristin Alexis Fergus, Elizabeth Storer, Moses Arinaitwe, Solomon Kamurari, Moses Adriko

**Affiliations:** 1 Firoz Lalji Insitute for Africa, London School of Economics and Political Science, London, UK; 2 Ministry of Health, Kampala, Uganda; 3 Uganda-UK Health Alliance, Kampala, Uganda

**Keywords:** COVID-19, Sub-national health workers, Information dissemination, Health communication, Infodemic, Rumours

## Abstract

**Background:**

In many places, health workers at the sub-national level are on the frontlines of disseminating information about coronavirus (COVID-19) to communities. To ensure communities are receiving timely and accurate information, it is vital health workers are kept abreast of the most recent recommendations, and guidance.

**Methods:**

An electronic survey was implemented to provide insights about the dissemination and utilisation of information and evidence related to the COVID-19 pandemic by health workers engaged at sub-national levels of the Ugandan health system. The aim of this survey was to provide insights about the dissemination and utilisation of information and evidence related to the coronavirus (COVID-19) pandemic by individuals engaged at sub-national levels of the health system.

**Results:**

Mass media and public health campaigns and outreach activities were deemed the most suitable means to reach communities with COVID-19 information. Given the reported disruption to public outreach campaigns, this is a particularly important consideration for the provision of information to communities. All materials should be adapted to the local context. The need for information on homecare of COVID-19 patients was highlighted, along with the need for updated local statistics as to COVID-19 cases to be relayed for health workers at sub-national levels.

**Conclusions:**

Understanding the sources of information used by health workers can facilitate the transfer of relevant and timely information, which in turn increases the use of such information by the Ugandan population. It is vital that these issues are continued to be monitored, and communication modes and content are actively responsive to the time- and place-specific needs of health workers and community members.

**Supplementary Information:**

The online version contains supplementary material available at 10.1186/s12913-021-07068-x.

## Background

Uganda’s adoption of the District Health System structure was formed largely through devolution in the late 1990s [1]. This structural strategy, which places decision-making at the district level, was endorsed at the WHO’s “Health for All” conference in 1987 as a way to achieve more equitable health care access by localised, adaptive approaches to intervention and service delivery [2]. In a similar vein, Uganda adopted the Community Health Worker programme in 2001, which was a formalised system of village health teams (VHTs), comprised of volunteers with a basic training in relevant health issues mandated with implementing community-based interventions [3]. Accordingly, Uganda’s response to the coronavirus (COVID-19) pandemic has been centred on the COVID-19 District Task Forces and District Rapid Response teams established in all 135 districts [4]. Guidance on COVID-19 activities, including those related to risk communication, are disseminated from the national level and are intended to be adapted to local, sub-district contexts [5].

As of September 1, 2021, Uganda had reported 99,408 confirmed cases and 3012 confirmed deaths due to COVID-19 [6]. The landscape of COVID-19 information has undergone rapid and substantial changes over the course of the pandemic. At times this has led to confusion, the spread of misinformation, and the public’s mistrust of scientists and health workers [7–9]. In many places, health workers at the sub-national level are on the frontlines of disseminating information about COVID-19 to communities. To ensure communities are receiving timely and accurate information, it is vital that health workers are kept abreast of the most recent recommendations and guidance. This is particularly important in light of the widespread dissemination of misinformation regarding COVID-19 causes and cures from unreliable sources, where health workers are in positions to potentially combat the inaccuracies and promote healthy behaviours. While previous work has shown that sub-national health workers are likely to rely on personal communications to inform their decision-making [10, 11], this has not been studied amongst sub-national health workers in the context of COVID-19 in Uganda specifically.

In this study, an electronic survey was implemented to provide insights about the dissemination and utilisation of information and evidence related to the coronavirus (COVID-19) pandemic by health workers engaged at sub-national levels of the Ugandan health system. In particular, the survey sought to understand: the sources of information health workers use for their work generally and for COVID-19 specifically; potential gaps in the formats and modes of dissemination of information to health workers; needs related to community and health facility COVID-19 information materials. The aim of this survey was to provide insights about the dissemination and utilisation of information and evidence related to the COVID-19 pandemic by individuals engaged at sub-national levels of the health system. The use of timely and accurate information about COVID-19 by health workers supports the population in risk reduction through community education and the adoption of protective behaviours.

## Methods

To address the objectives above, survey questions were developed to reflect the context of COVID-19 in Uganda. In total, there were 37 questions comprised of multiple choice and text answers (see Supplementary Materials for the survey questions) in the covering the following categories: demographic and professional details; sources for evidence and information about health generally and COVID-19 specifically; dissemination of health information into the communities; the state of COVID-19 and response in Uganda; COVID-19 myths and rumours.

The survey was written in and implemented using Qualtrics Survey software (version 2020, Provo, UT, USA). Both the survey questions and implementation were pre-tested with 50 individuals who were purposively selected across varying geographic, epidemiological, and health system levels. For the analyses, the responses were downloaded into Microsoft Excel and matched with the original sampling frame to confirm demographic and employment details. Analyses were conducted using NVIVO (version 12.6.0), Stata SE (version 15.1), and R (version 1.3.1073.) Maps were created using QGIS (version 3.4.15-Madeira.)

### Sample

Following the district health system structure in Uganda, the sample included district health officers (DHOs), district vector control officers (DVCOs), health workers from lower health facilities, and members of village health teams (VHTs) from Uganda’s 135 districts. The sample was purposively constructed by the Ministry of Health – Vector Control Division through snowball sampling using district-level contacts (DHOs and DVCOs), and consisted of 410 individuals. To facilitate the electronic survey, a master email and mobile contact list of the sample was generated.

### Implementation

The electronic survey was developed using Qualtrics survey software in both a mobile- and web-friendly formats. An introduction email, including a participant information form and a link to the survey, was sent to the sample of potential participants. The link directed potential participants to an electronic Informed Consent page, which could be signed electronically and was required to continue onto the survey questionnaire. Participants who were unable to access the Qualtrics survey or fully complete the survey online due to mobile data access issues were offered a phone survey option, where a researcher would read the survey questions and record the responses in the software, as an alternative. This method had been pre-tested with 10 individuals of varying internet connectivity for this survey. The individuals who participated via the phone method were read the Participant Information and Informed Consent forms, and verbal consent was received and recorded within the Qualtrics survey software. To encourage survey participation, follow-up measures included four email reminders, three mass SMS alerts, at least one phone call and SMS or Whatsapp message to the entire sample of potential participants. The survey implementation began on 3rd December 2020 and closed on 15th January 2021.

## Results

### Survey participation

**Fig. 1:**
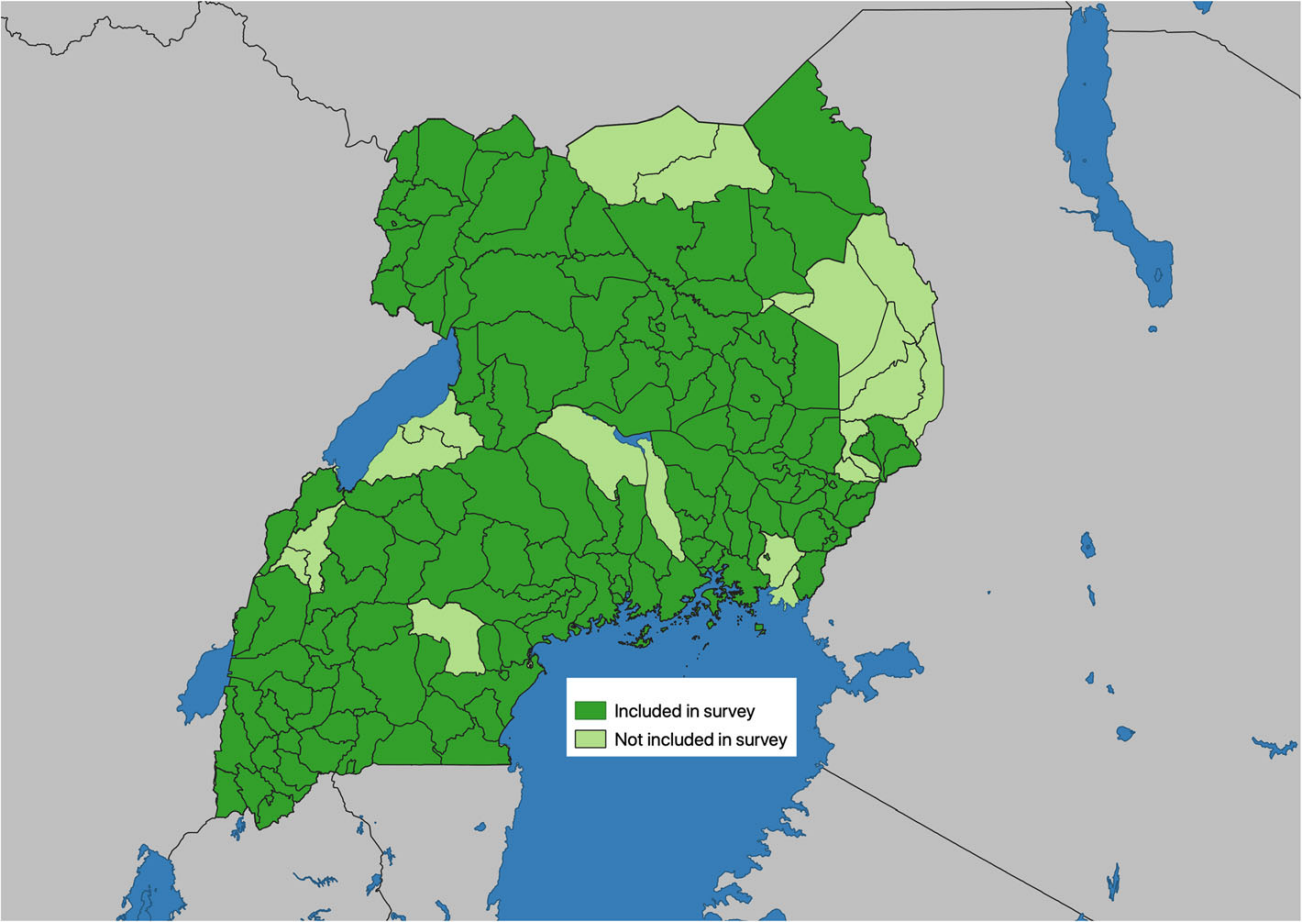
Map of district included in the survey results. Note: This map was created using QGIS (version 3.4.15-Madeira; https://www.qgis.org/)

The response rate was 55% (*n* = 225) across the sample, with 197 participants who completed the survey online and 28 participants who elected to respond via phone call. This response rate was higher than expected compared to other electronic surveys, which have reported response rates of approximately 25% (for example [12, 13].) Respondents participated from 84% (*n* = 113) of districts in Uganda (Fig. 1)

### Respondent characteristics

The distributions of respondent characteristics are shown in Table 1. Most of the respondents have more than 5 years of professional experience (91%) and are involved with programme implementation (65%). In terms of education, most of the respondents had achieved at least a Diploma (88%) and only eight individuals (4%) reported that they had not completed a minimum of a Certificate qualification.

**Table 1:** Survey respondent characteristics

Characteristic	n	%
*Professional experience (yrs)*		
< 1	2	1
1–5	18	9
6–10	58	29
11–20	72	36
20	52	26
*Education*		
Primary	2	1
Secondary	6	3
Certificate	17	8
Diploma	65	32
Bachelor’s degree	72	36
Master’s degree	38	19
Doctorate	1	0.5
*Region*		
Central	34	17
Eastern	67	33
Northern	61	30
Western	39	19
*Job activities*^a^		
Policy development	11	5
Programme implementation/support	131	65
Clinical	86	43
Laboratory	14	7

^a^Respondents could select more than one activity

### Perspectives on the state of COVID-19 in Uganda

To provide context to the responses, the survey captured the respondents’ perspectives on the state of COVID-19 in Uganda. The majority of participants reported that Uganda’s response had been either “Good” or “Fair” compared to other countries (49 and 31%, respectively.) Further, in terms of the national COVID-19 guidelines at the time of the survey, 79% of respondents “Agreed” or “Strongly Agreed” that they were clear and sufficient for the general population.

**Fig. 2:**
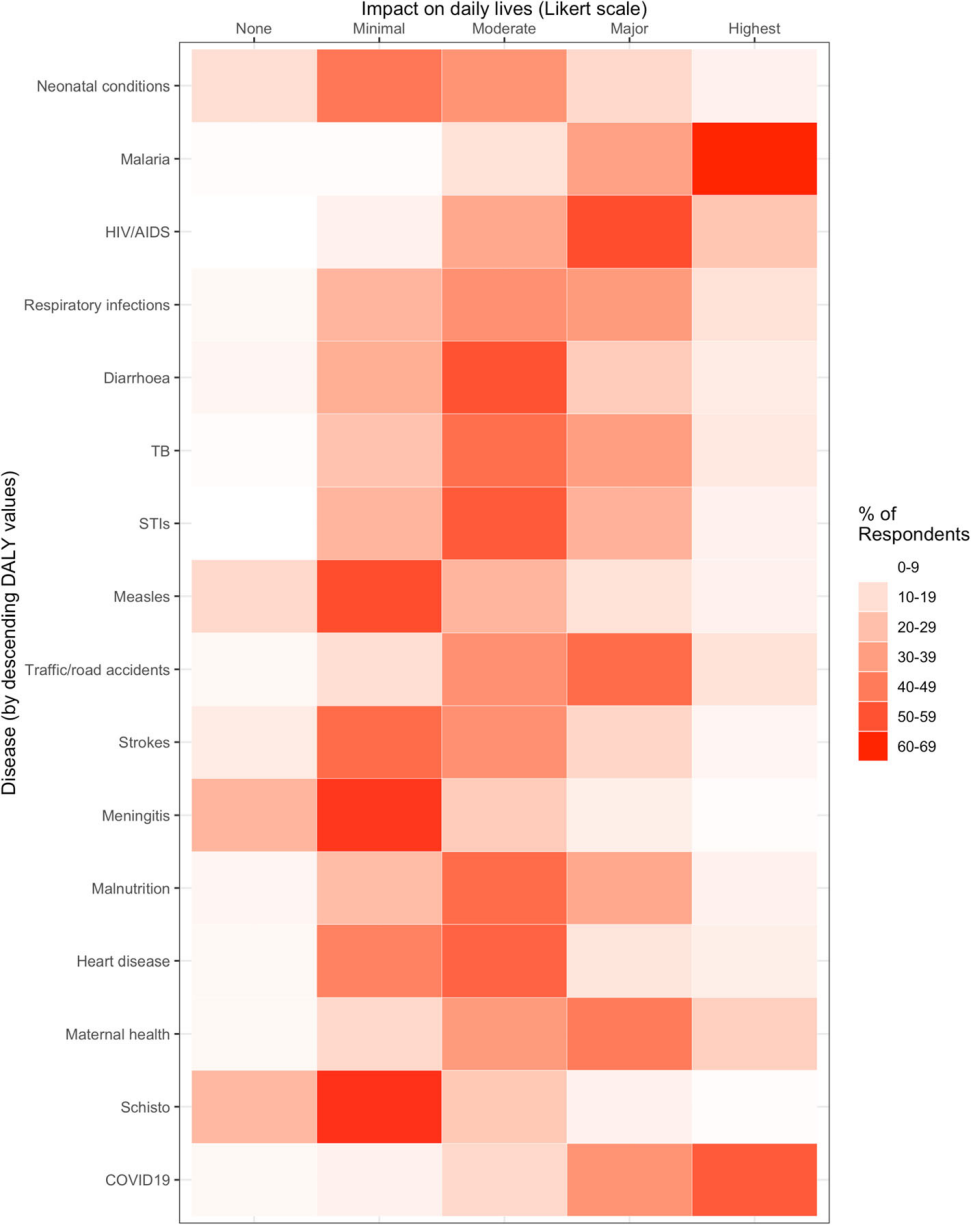
Rating the burden of diseases on the everyday lives of Ugandans

To understand how the burden of COVID-19 compares to the impact of other diseases on the everyday lives of Ugandans, survey participants were asked to rate COVID-19 alongside 15 diseases with the highest Disability Adjusted Life Years (DALYs) in Uganda, as reported by the Global Burden of Disease estimates for the year 2019 [14]. Figure 2 shows the percentage of respondents who rated the burden of each disease from “No impact” to “Highest impact” on the everyday lives of Ugandans, with the diseases listed in descending order from highest DALYs to lowest DALYs. COVID-19 is listed last because DALY estimates are not available yet. Most participants (over 60%) described malaria as having the “highest impact” on Ugandans. Over 50% of respondents described neonatal conditions as having “no impact” or a “minor impact” on the lives of Ugandans, while only 3% described neonatal diseases as the “highest impact”, despite this being the highest cause of DALYs in Uganda.

**Fig. 3:**
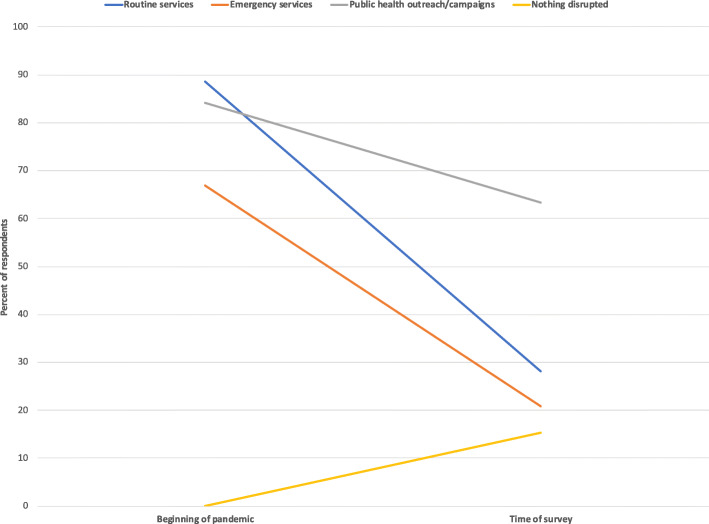
Percent of respondents who reported disruptions across health services at the beginning of the COVID-19 pandemic and at the time of survey (approx. 9–12 months later) (*n* = 225)

Reports of disruptions to health services were widely reported at the beginning of the COVID-19 pandemic [15, 16]. The participants were asked to select which of the services had been disrupted at the beginning of the pandemic and then also select which services were still disrupted at the time of the survey (approximately 9–12 months after the pandemic began.) All respondents reported that at least one type of health service had been disrupted at the beginning of the pandemic, while over 14% of respondents reported that there were no longer disruptions to any health services at the time of the survey. Figure 3 shows the percentage of participants who responded that routine services, emergency services, and/or public health outreach and campaigns had been disrupted at the two time points. At the time of the survey, the majority of respondents still thought public health outreach/campaigns were still disrupted, while most respondents felt that there were no longer disruptions to routine and emergency services. When asked to name specific programmes or services that were most urgently needed to restart, the most frequently mentioned were HIV outreach services, antenatal services, and immunization campaigns.

Personal protective equipment (PPE) is a key aspect of limiting the transmission of COVID-19. The survey participants were asked about whether they had adequate PPE in order to be protected during their work activities. In total, 85% of respondents did not have adequate PPE to protect themselves during work activities. The percentage did not vary significantly by job level. However, there were variations geographically, with districts in the Central and Northern regions reporting more adequate PPE compared to those districts in the Western and Eastern regions (Table 2.)

**Table 2:** Percent of respondents reporting that they do not have adequate PPE to protect themselves during work activities

Geographic region	n	%
All regions	191	85
Central	173	77
Eastern	202	90
Northern	175	78
Western	214	95

When asked to report which specific PPE they were lacking, the respondents reported the following: 55% lacking surgical masks, 69% lacking respirator masks, 44% lacking gloves, 68% lacking face shields/visors, 63% lacking eye goggles, and 73% lacking gowns, aprons, or coveralls.

### Sources of information for health workers

Respondents were asked to select the information sources they use or access at least once per month (Table 3) The results indicate a decidedly social process of information gathering, with three of the five most utilised sources being modes of interacting with others. The second most frequently selected source of information was hard copies of Uganda Ministry of Health materials, which was reiterated throughout the survey. This is especially key for those individuals, particularly those working at the sub-district level, who may not have reliable access to the internet to find and use the most up-to-date materials on websites. Interestingly, peer-reviewed articles and reports from outside Uganda were reportedly used least frequently by respondents.

**Table 3:** Percent reporting use of specific sources of information for their job

Sources of information	n	%
Social Media	160	79
MoH hard copy materials	148	73
Work meetings/colleagues	147	73
MoH electronic materials	129	64
Community meetings	128	63
WHO electronic materials	109	54
Mass media	104	51
Textbooks	102	51
WHO hard copy materials	101	50
Published papers and reports from Uganda	86	43
Religious leaders	86	43
Published peer-reviewed articles and reports from outside Uganda	68	34

In terms of sources consulted specifically for COVID-19 information, the respondents were asked which current channels utilised by the Ministry of Health they used to access information. The most frequently reported source of information was mass media/news media websites, followed by the Uganda Ministry of Health website/media (Table 4)

**Table 4:** Percent reporting use of channels for COVID-19 information for their job

Channel	n	%
Mass/news media	180	89
MoH website/media	156	77
Social media	140	69
Personal messaging apps	127	63
WHO website/media	120	59
Peer-reviewed journals	56	28

About three-quarters (74%) of respondents reported that they had adequate information about COVID-19 to stay safe and perform their jobs. When asked to specifically identify how the COVID-19 information they receive could be improved, the responses fell into four categories: improvements in the actual content and format information, increased training and learning opportunities, improvements in the dissemination strategies, and empowering health workers. To allow for prioritisation of improvements, the distribution of respondents who reported suggestions under each of these categories is shown in Table 5, along with examples of the most mentioned suggestions under each category.

**Table 5:** Respondents’ suggested improvements in the COVID-19 information they receive

Responses	Examples	n	%
Improve the content and format of COVID-19 information	Translated versions of materials, add visual content to materials, share current research findings	60	46
Training and learning opportunities for health workers	Online workshops, mentorships, training follow-ups, training refresher courses	46	35
Improve the dissemination of COVID-19 information	Hard copies of guidance/information, utilisation of multiple forms of communication (especially SMS and email), more frequent updates	42	31
Health worker empowerment	Co-development of guidelines and programmes, empowering lower levels of health service deliver, motivating heal workers	22	17

To understand the potential impacts of having adequate information, the respondents were asked about the extent to which they were responsible for disseminating COVID-19 information to others. The vast majority of respondents reported that they were responsible for disseminating accurate and timely information about COVID-19 to various groups (Table 6) This highlights the importance of ensuring that the information sent to health workers in the first place is adequate and timely, as it a part of a larger chain of information dissemination that has significant impacts on combatting COVID-19 in Uganda.

**Table 6:** Percent of respondents who report being responsible for disseminating COVID-19 information to various groups

Group	n	%
Community members	186	91
Other health workers	168	88
Patients	148	77
Policymakers	111	58
Other government officials	4	2

### Sources of information in the communities

**Fig. 4:**
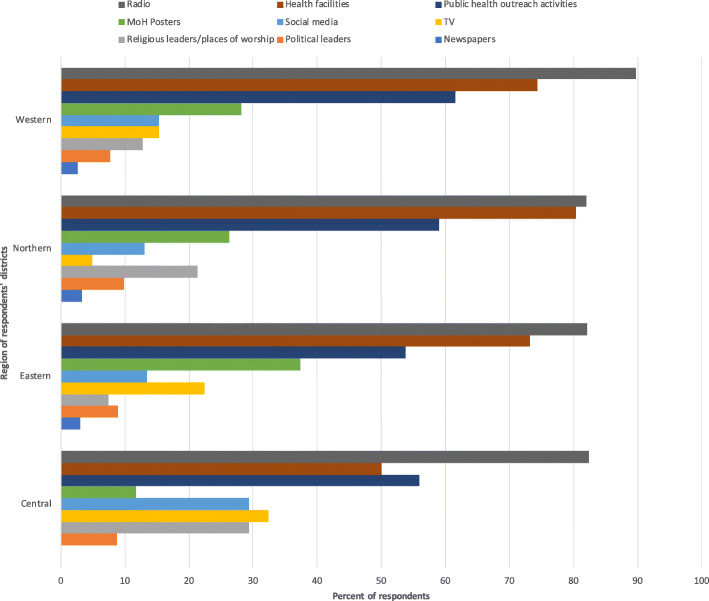
Most used sources for health information by communities

In order to efficiently and effectively target health information to the communities, the survey asked respondents for their insights as to how communities receive information about health in general and COVID-19 specifically. Figure 4 shows where individuals in the communities receive information about health generally. The most frequently selected source of information was the radio across districts in all regions.

In terms of COVID-19 specifically, as shown in Table 7, mass media and public health campaigns and outreach activities were mentioned as the most effective ways to disseminate COVID-19 information in open-ended answers by the majority of survey participants (Table 7). The latter is especially important, given that the majority of respondents also reported that public health campaigns and outreach activities were still disrupted (Fig. 3). Additionally, approximately one-third of respondents mentioned the display of COVID-19 information in public places and during social and community activities. This would require the production of materials in hard copies, which is something that district- and sub-district level respondents highlighted as a particular need throughout the survey.

**Table 7:** Most effective ways to disseminate COVID-19 information to the communities, as mentioned by % of respondents

Response types	Examples	n	% of respondents
Mass media	Newspapers, TV, radio	115	60
Public health campaigns and outreach activities	Community-based campaigns, public address system, community sensitisation, home-to-home visits, town criers, focus groups	111	58
During community and social activities	Churches, community meetings, funerals, schools	73	38
Materials displayed in public places	Materials, leaflets, posters, brochures	58	30
In communications from leaders	Community leaders, District leadership, police, political leaders, religious leadership	50	26
Community-based health workers	CHWs, VHTs	50	26
Routine health services	Health centres, health education, head education	40	21
Through translated materials	Materials in local languages	8	4
Social media	Facebook, Twitter	6	3
Telephone dissemination	Telephone calls or SMS	2	1
Transportation	Boda Boda drivers, public transportation	2	1

**Fig. 5:**
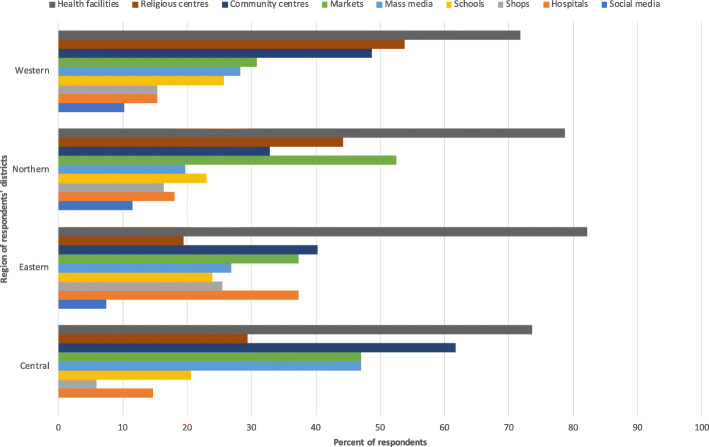
Places most in need of additional COVID-19 information in the communities

Ninety-six percent of respondents reported that additional COVID-19 informational materials were needed in the communities. Respondents selected the top three places most in need of additional materials (Fig. 5), with radio selected most often across the regions.

The next most frequently selected places to display information were religious centres in districts in the Western region, markets across districts in the Northern region, and in community centres in the Central and Eastern districts.

In terms of content of this additional information, the most frequently mentioned need was specific and detailed information on proper homecare of COVID-19 cases. Respondents also highlighted the need for current local statistics, risk behaviours relevant to the area, and guides to proper use of PPE and disinfectants. Over half of the survey participants reported that information in hard copy/paper format and translated materials were needed to disseminate COVID-19 information into the communities, which was identified throughout the survey.

In terms of target groups, young people were most often mentioned, followed by the elderly and school groups. Local political leaders and traders were also frequently mentioned as important target groups for additional information. These groups are all different in terms of their communication methods and COVID-19 risk behaviours, and therefore efforts should be made to tailor the COVID-19 informational materials for each group.

### Rumours and myths

Uganda’s COVID-19 response is delivered to villages which feature a plurality of public authority, empowering religious leaders to deliver public health messaging. Our results indicated the prevalence of religious explanations for COVID-19, and the involvement of Christian and Islamic leaders, as well as traditional/ clan elders in the response. Approximately one-third (*n* = 87) of respondents reported hearing of religious explanations, which included beliefs derived from Christianity, Islam, or customary religious practices, in relation to COVID-19. The prevalence of religious explanations was higher across districts in the Eastern (47%) and Northern (43%) regions compared to the Central and Western regions. Across all respondents, 33% of religious explanations related to COVID-19 being caused by divine will – manifesting as a punishment from a Christian or Muslim God, or signalling the “end of the world”. For example:

“The disease was already proclaimed by God and it truth has come”

“Christians say that the Bible says that this world has come to an end, and for this to happen many signs will manifest such as diseases without cure, and now there is COVID”

“COVID-19 is a God related punishment for the sins of mankind.”

Twenty-nine percent attributed COVID-19 prevention, or cure, to Christian or Muslim practices, including prayer, or the cessation of public worship practices. For example:

“Communal burials, marriage introductions and trans-night church prayers spread COVID-19”

“God heals all diseases so stopping church services worsens the disease”.

**Fig. 6:**
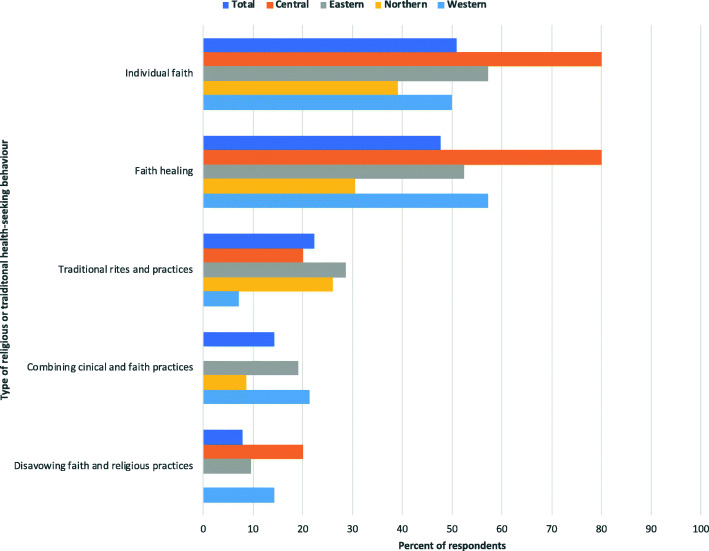
Percent of respondents reporting types of religious or traditional health-seeking behaviours, by total and geographic region

The respondents were also asked to describe any COVID-19 health- or care-seeking behaviours based in religious beliefs (Fig. 6).

These results thus point to emerging local conversations and attempts to define the role of religious and traditional practitioners in the COVID-19 response. Interestingly, when asked specifically about religious explanations for COVID-19, the respondents identified multiple connections to Christianity and Islam, along with traditional forces. Yet, when asked generally about rumours and myths, a much broader rumourscape was reported, which included other forms of healing, substances and global fears of bio-terrorism and capitalism exploration.

International experts have drawn attention to analysing the form and production of misinformation relating to COVID-19 [17]. Rumours and myths, spread both online and offline, could present a problem for the effective implementation of public health measures. COVID-19 is the first pandemic where media and social media have been used at such a massive scale to disseminate government guidance and safety information to populations. Yet, at the same time, these channels are being used to amplify misinformation related to the virus. The WHO has termed this abundance of false and misleading information as an “infodemic”, and has suggested that combatting misinformation is integral to the COVID-19 response [17]. The following section outlines the contours of the infodemic in Uganda, as reported by survey respondents.

The respondents reported widespread rumours about myths, origin, protection and cures for COVID-19. Identifying the channels through which rumours spread is an essential step in combatting the “infodemic,” which has accompanied the spread of COVID-19. In order to understand the production of rumours and myths and target response efforts, respondents were also asked to identify the sources of misinformation.

Table 8 shows the frequency and distribution of sources reported by the survey participants. Community talk and social media were identified as the most prevalent sources of rumours. Secondary to this, political and religious leaders were identified as figures which incited rumours about COVID-19. Responses indicate that sources of rumours are both offline and online. Whilst social media was identified as a key medium through which misinformation was spread, respondents also identified sources as being in communities, or leadership.

**Table 8:** Frequency and distribution of responses on the sources of rumours, as reported by > 5% of respondents

Type of source	n	%
Community talk/gossip	88	46
Social Media	77	40
Politicians or Political Leaders	33	17
Religious Leaders	17	9
Peers	12	6

These results suggest that plans to combat misinformation must take a dual focus: working with communities and leaders influential in said communities, as well as analysis patterns of use and access to social media. Further research should identify how online sources are interpreted and spread through community networks.

## Discussion

At the time of the survey, the majority of respondents still thought public health outreach/campaigns were being disrupted due to the focus of health providers on COVID-19, though most believed that disruptions to routine and emergency services had ended. When asked to name specific programmes or services that were most urgently needed to restart, the most frequently mentioned were HIV outreach services, antenatal services, and immunization campaigns.

With regards to health workers’ sourcing of information, the most frequently selected source was social media, followed by meetings and colleagues. This illustrates this importance of creating opportunities for knowledge exchange amongst health workers themselves. The most frequently reported source of information was mass media/news media websites, followed by the Uganda Ministry of Health website/media. Health workers also consulted WHO information for guidance on COVID-19. When asked to specifically identify how the COVID-19 information they receive could be improved, the responses fell into four categories: improvements in the actual content and format information, increased training and learning opportunities, improvements in the dissemination strategies, and empowering health workers.

Mass media and public health campaigns and outreach activities were deemed the most suitable means to reach communities with COVID-19 information. Given the reported disruption to public outreach campaigns by our respondents, this is a particularly important consideration for the provision of information to communities. Respondents highlighted the utility of public displayed posters in social and communal spaces, as well as for materials translated into local languages. The need for hard copy health information was highlighted throughout the survey as being important for the dissemination of health information. The most important place to display information across regions was health facilities, including health centres and hospitals. Religious centres and markets were also noted as important places to display COVID-19 guidance to communities. As above, this material should be adapted to the local context. The need for information on homecare of COVID-19 patients was highlighted, along with the need for updated local statistics as to COVID-19 cases to be relayed for health workers at sub-national levels.

Religious explanations relating to COVID-19 origins, protections and cures are prevalent across Uganda at the sub-national level. Such explanations are diverse, drawing on beliefs connected to Christianity, Islamic and traditional knowledge. These explanations may affect adherence to public health measures, yet scepticism exists as to the validity of alternative cures. Rumours relating to the origins, existence and cures for, COVID-19 exist across Uganda. Rumours draw on diverse logics, involving ideas about geopolitics, religious, politics, and herbal traditions. Understandings of ‘African immunity’ have been superseded by rumours regarding cures as the pandemic has progressed. Rumours were most commonly spread through community talk and through social media.

There are several limitations to the results presented here. The survey design included purposive sampling; therefore, the results are not representative of all health workers at the sub-national level in Uganda. Similarly, responses were not collected from all districts and therefore conclusions cannot be drawn about all administrative area. Given that this was an electronic survey, individuals without mobile network access may not have been able to participate, even with the accommodations provided by the researchers. Finally, given the changing nature of the COVID-19 pandemic and responses, it is important that the results are interpreted within the time- and place-specific features of the survey data collection.

## Conclusion

The COVID-19 pandemic continues to be a burden on Uganda’s health system. The use of timely and accurate information about COVID-19 by health workers support risk reduction in the population through the dissemination of this information and the adoption of protective behaviours. Specifically, understanding the sources of information used by health workers can facilitate the transfer of relevant and timely information, which in turn increases the use of such information by the Ugandan population. It is vital that these issues are continued to be monitored, and communication modes and content are actively responsive to the time- and place-specific needs of health workers and community members.

## Supplementary Information

**Additional file 1.**

## Data Availability

The dataset collected and analysed during the current study is available from the corresponding author on reasonable request.

## References

[1] WHO (1987). Declaration on strengthening district health systems based on primary health care.

[2] Henriksson DK, Ayebare F, Waiswa P, Peterson SS, Tumushabe EK, Fredriksson M (2017). Enablers and barriers to evidence based planning in the district health system in Uganda; perceptions of district health managers. BMC Health Serv Res.

[3] Musoke D, Ndejjo R, Atusingwize E, Ssemugabo C, Ottosson A, Gibson L (2020). Panacea or pitfall? The introduction of community health extension workers in Uganda. BMJ Global Health.

[4] Sarki AM, Ezeh A, Stranges S (2020). Uganda as a role model for pandemic containment in Africa. Am J Public Health.

[5] WHO Africa. Uganda to win or lose COVID-19 war in communities: World Health Organization - Regional Office for Africa; 2020. https://www.afro.who.int/news/uganda-win-or-lose-covid-19-war-communities. Accessed 19 Apr 2021

[6] WHO. Uganda: WHO Coronavirus disease (COVID-19) dashboard with vaccination data. https://covid19.who.int. Accessed 2 Sept 2021.

[7] Jaiswal J, LoSchiavo C, Perlman DC (2020). Disinformation, misinformation and inequality-driven mistrust in the time of COVID-19: Lessons unlearned from AIDS denialism. AIDS Behav.

[8] Ventola CL (2014). Social media and health care professionals: benefits, risks, and best practices. Pharm Ther.

[9] Alvarez-Risco A, Mejia CR, Delgado-Zegarra J, Del-Aguila-Arcentales S, Arce-Esquivel AA, Valladares-Garrido MJ (2020). The Peru approach against the COVID-19 Infodemic: insights and strategies. Am J Trop Med Hyg.

[10] Bulthuis SE, Kok MC, Amon S, Agyemang SA, Nsabagasani X, Sanudi L (2021). How district health decision-making is shaped within decentralised contexts: a qualitative research in Malawi, Uganda and Ghana. Global Public Health.

[11] Kumar MB, Taegtmeyer M, Madan J, Ndima S, Chikaphupha K, Kea A (2020). How do decision-makers use evidence in community health policy and financing decisions? A qualitative study and conceptual framework in four African countries. Health Policy Plan.

[12] Rees CA, Lukolyo H, Keating EM, Dearden KA, Luboga SA, Schutze GE (2017). Authorship in paediatric research conducted in low- and middle-income countries: parity or parasitism?. Tropical Med Int Health.

[13] Murunga VI, Oronje RN, Bates I, Tagoe N, Pulford J (2020). Review of published evidence on knowledge translation capacity, practice and support among researchers and research institutions in low- and middle-income countries. Health Res Policy Syst.

[14] IHME (2020). Global burden of disease study 2019 (GBD 2019) results.

[15] WHO AFRO. WHO | Regional Office for Africa: Easing COVID-19 impact on key health services; 2020. https://www.afro.who.int/news/easing-covid-19-impact-key-health-services. Accessed 25 Apr 2021

[16] Ponticiello M, Mwanga-Amumpaire J, Tushemereirwe P, Nuwagaba G, King R, Sundararajan R (2020). “Everything is a Mess”: How COVID-19 is impacting engagement with HIV testing services in Rural Southwestern Uganda. AIDS Behav.

[17] WHO (2020). Managing the COVID-19 infodemic: Promoting healthy behaviours and mitigating the harm from misinformation and disinformation.

