# Supplementary material for: COVID-19 information dissemination in Uganda: Perspectives from sub-national health workers
Source: BMC Health Serv Res. 2021 Oct 7;21:1061. doi: 10.1186/s12913-021-07068-x (PMC8496434; doi:10.1186/s12913-021-07068-x)
Supplement: Supplementary file 1 — Additional file 1. [file 12913_2021_7068_MOESM1_ESM.docx]

**COVID-19 Information Dissemination in Uganda: Perspectives from sub-national health workers**

**Supplementary Materials**

**Full Survey Questions Draft**

Section 1. Demographic and employment

1. Please select the level of focus which best describes your work:
   1. National level policy and/or practice
   2. District level policy and/or practice
   3. Sub district health services implementation
   4. Village level health service provision
   5. Others, Please specify
2. Please select which of the following best describes the sector of your current employment:
   1. Private
   2. Public (MoH)
   3. NGO, non-profit, charity, foundation
   4. Other, please specify:
3. Which of the following categories best describes the nature of your current job? (check all that apply)
   1. Policy development
   2. Programme implementation
   3. Clinical activities
   4. Laboratory activities
   5. Other, please specify:
4. How many years of professional experience do you have?
   1. <1 year
   2. 1-5 years
   3. 6-10 years
   4. 11-20 years
   5. >20 years
5. What is the highest level of education you have achieved?

a. Primary

b. secondary

c. certificate

d. Diploma

e. Bachelors

f. Masters degree

g. Doctorate degree (PhD, MD)

h. Other, please specify:

1. Please select the district where your work is based (this may be different from where you live):

[drop down list]

1. Please select the district where you live (this may be different from where you work):

[drop down list]

Section 2. Evidence and information for your job

The following questions ask about where you get information for decision-making in your current job. These are general questions to provide background that allow us to assess which information sources are most effective.

1. Do you regularly (at least 1 time per month) use any of the following sources of information in your work? Please check all that apply:
   1. Uganda Ministry of Health website or electronic publications
   2. Uganda Ministry of Health hard copy publications (including posters or pamphlets)
   3. World Health Organization website or electronic publications
   4. World Health Organization hard copy publications (including posters and pamphlets)
   5. Journal articles/reports from sources in Uganda
   6. Journal articles/reports from sources outside of Uganda
   7. Textbooks
   8. Social media
   9. Colleagues
   10. Community members
   11. Religious leaders
   12. Online news websites
2. How often do research findings affect the decisions you make in your current job?
   1. Never
   2. Rarely
   3. Sometimes
   4. Often
   5. Always
3. In the past, have you had problems using peer-reviewed journal articles? (Please check all that apply):
   1. Yes, they required payment
   2. Yes, the internet was too slow or crashed
   3. Yes, it was difficult to find relevant articles
   4. No, I have tried to access and not had any problems
   5. No, I have not tried to access
4. How often do the following influence the decisions you make in your work? (Never, Rarely, Sometimes, Often, Always)
   1. Personal experience
   2. Directives from your supervisor
   3. Opinions from community members
   4. Politicians
   5. Results from peer-reviewed journal articles
   6. WHO guidelines
   7. MOH guidelines
   8. Conversations with family and friends
   9. News media (TV, radio, newspapers)
   10. Budget or funding availability
   11. National-level strategic plans

Section 3. Community/population health and health information

1. Where do most people in your community get information about health? (Please select the top three sources of information)
   1. TV
   2. Radio
   3. Posters
   4. Public ealth outreach campaigns (e.g. megaphones, sensitization campaigns)
   5. Health facilities, clinics, hospitals
   6. Social media
   7. Newspapers
   8. Political leaders
   9. Religious leaders, places of worship
2. Based on your knowledge and experience, as of TODAY, please rate the burden each of the following health-related issues places on the everyday lives of Ugandans, from low impact (1) to a high impact (5):
   1. Malaria
   2. HIV/AIDS
   3. Sexually Transmitted Infections (STIs, other than HIV/AIDS)
   4. Tuberculosis
   5. Heart disease
   6. Strokes
   7. Traffic/road accidents
   8. COVID-19
   9. Diarrhoea
   10. Schistosomiasis/bilharzia
   11. Meningitis
   12. Malnutrition
   13. Maternal health in pregnancy or birth
   14. Neonatal disorders
   15. Measles
   16. Respiratory infections

Section 4. COVID-19 questions

1. Compared to other countries, how would you describe the response to the COVID-19 pandemic in Uganda?
   1. Excellent
   2. Good
   3. Fair
   4. Poor
   5. Very poor
2. To what extent do you agree with the following statement: “The national COVID-19 guidelines are clear and sufficient for the general population.”
   1. Strongly agree
   2. Agree
   3. Neutral/undecided
   4. Disagree
   5. Strongly disagree
3. Where do you obtain information on COVID-19? (Please check all that apply)
   1. Official international health organisation websites and media
   2. Official government websites and media
   3. News media (TV, radio, magazines, newspaper)
   4. Social media (Twitter, Instagram, Facebook)
   5. Personal messaging apps (Whatsapp, SMS)
   6. Journals
   7. Other, please specify:
4. Do you feel that you have received adequate information and guidance on how to deal with COVID-19 in your job?
   1. Yes
   2. No
   3. Other, please explain:
5. How could the information and guidance provided to you could be improved?

[free text answer]

1. Do you currently have adequate personal protective equipment (PPE) against COVID-19 in your current job?
   1. Yes
   2. No
   3. Other, please explain:
2. Please check which of the following personal protective equipment you need in your current job, yet you **do not** have access to:
   1. Surgical masks
   2. gloves
   3. face shields/visors
   4. eye goggles
   5. medical gowns
   6. aprons
   7. respirator masks
   8. Other, please specify:
   9. Not lacking any ppe
3. Does the general population have adequate access to appropriate personal protective equipment (e.g. masks, gloves) again COVID-19?
   1. Yes
   2. No
   3. Other, please explain:
4. Are you responsible for dealing directly with potential or active COVID-19 cases?
   1. Yes
   2. No
   3. Other, please specify:
5. Are you responsible for providing COVID-19 information or guidance to any of the following groups? (Please check all that apply)
   1. Patients
   2. Other health workers (e.g. in trainings or to subordinates)
   3. Out in the community
   4. Policymakers (e.g. giving presentations at Task Force meetings)
   5. Others, please specify:
6. Based on your knowledge and experiences, what are the most effective ways to provide information about COVID-19 to communities?

[free text answer]

1. What are the three most important places to display COVID-19 information?
   1. Health centres
   2. Hospitals
   3. Shops
   4. Social media posts
   5. Mass media (TV, radio) adverts
   6. Markets
   7. Schools
   8. Religious centres
   9. Community centres
   10. Other, please specify:
2. Do you think additional COVID-19 informational materials are needed at the community level across Uganda?
   1. Yes
   2. No
   3. Other, please explain:
3. Specifically, which additional materials about COVID-19 should be provided in the communities and which specific groups should be targeted?

[free text answer]

1. **During the initial response to COVID-19 in Uganda**, were any of the following disrupted? Please check all that apply:
   1. Routine health services
   2. Emergency health services
   3. Public health campaigns (such as
   4. Other, please explain:
2. **As of today**, are any of the following being disrupted due to covid-19? Please check all that apply:
   1. Routine health services
   2. Emergency health services
   3. Public health campaigns (such as
   4. Other, please explain:
3. Out of everything that has been disrupted due to COVID-19, in your district, which health-related programmes, services, or interventions are in most urgent need of starting or expanding again?

[free text answer]

Section 5 traditions, religious, myths section

1. Have you heard of any religious explanations which accompany the spread of COVID-19?
   1. Yes
   2. No
2. [*If yes in Q28] Please describe any religious explanations you have heard.

[free text answer]

1. Have you heard of any health practices based in religious beliefs?
   1. Yes
   2. No
2. [*If yes in Q30] Please describe any health seeking behaviours based in religious beliefs.

[free text answer]

1. What are the most widespread rumours and myths about the origin, protection, or cures for COVID-19?

[free text answers]

1. What are the main sources for rumours and myths about COVID-19?

[free text answers]

1. What are the top three things that could be done to improve Uganda’s fight against COVID-19?
   1. First improvement__
   2. Second improvement__
   3. Third improvement__
